# Implicit and explicit sense of agency in individuals with obsessive-compulsive tendencies

**DOI:** 10.3389/fpsyg.2025.1664222

**Published:** 2025-12-05

**Authors:** Stefan Schmidt, Gerd Wagner

**Affiliations:** 1 Department of Psychiatry and Psychotherapy, Jena University Hospital, Jena, Germany; 2 Center for Intervention and Research on Adaptive and Maladaptive Brain Circuits Underlying Mental Health (C-I-R-C), Jena-Magdeburg-Halle, Germany

**Keywords:** obsessive-compulsive disorder, sense of agency, temporal binding, sensorimotor theory, trait anxiety, OCI-R

## Abstract

**Introduction:**

The sense of agency (SoA), or the perception of control over one’s actions and their outcomes, has been proposed to be attenuated in individuals with obsessive–compulsive disorder (OCD) and obsessive–compulsive (OC) tendencies. According to the Sense of Agency and Seeking Proxies for Internal States (SPIS) model, individuals with higher OC tendencies (HOC) may exhibit an altered SoA characterized by diminished tone binding but heightened action binding compared to individuals with lower OC tendencies (LOC).

**Methods:**

To investigate this, 29 healthy participants completed a temporal binding task to measure implicit SoA, alongside self-report measures assessing OC symptoms, anxiety, depression, and explicit SoA. Participants were divided into HOC and LOC tendency groups based on median OCI-R scores. Group comparisons and regression analyses were conducted to examine associations between SoA measures and clinical traits.

**Results:**

Significant action and tone binding effects were observed across the entire sample, indicating a robust implicit SoA. Contrary to our hypotheses, no significant differences in action or tone binding effects were found between HOC and LOC groups, suggesting a preserved implicit SoA in individuals with higher OC tendencies. Regression analyses revealed that trait anxiety, rather than OC symptom severity, significantly predicted tone binding in the whole sample, indicating an attenuated SoA with increasing trait anxiety levels. Within the HOC group, OC symptom severity significantly predicted tone binding, challenging the SPIS model’s assumption that individuals with high OC tendencies would exhibit diminished tone binding effects. Despite preserved implicit SoA, explicit measures revealed significant group differences, with HOC individuals reporting a lower global SoA.

**Discussion:**

These findings suggest that while implicit, sensorimotor aspects of SoA remain intact in individuals with OC tendencies, alterations emerge at the explicit, cognitive reflective level. This dissociation may reflect compensatory control processes or cognitive biases rather than primary deficits in action–outcome processing. Our findings underscore the need for further research in clinical OCD populations to disentangle the effects of OC symptom severity and subtype on SoA. These insights contribute to a nuanced understanding of agency distortions in compulsive behavior.

## 1 Introduction

Obsessive-compulsive disorder (OCD) is characterized by either obsessions, compulsive rituals or, in most cases the occurrence of both (APA, 2013). Obsessions are thoughts, ideas, and concepts that occupy a person in an intrusive manner leading to strong unpleasant emotions, such as anxiety or disgust. Compulsions, on the other hand, are repetitive behaviors that are performed and constantly repeated, often but not exclusively, as a response to obsessions. Affected individuals are extremely restricted in their daily lives due to the disorder and spend several hours dealing with their obsessions and compulsions. In addition, patients suffering OCD are often experiencing loss of control over their own thoughts and actions on the one hand and on the other hand, feel a strong need for controlling their environment to an excessive degree in order to prevent negative outcomes. This phenomenological description of obsessions and compulsions points to an altered sense of agency (Schmidt et al., 2021). The sense of agency is the “experience of controlling our own actions, and through them, events in the outside world” (Haggard, 2017). To date, only a few studies have investigated the apparently disturbed sense of agency in patients with OCD or unaffected subjects with obsessive-compulsive (OC) tendencies (Gentsch et al., 2012; Oren et al., 2019).

In recent years, cognitive-behavioral and metacognitive theories have received considerable attention in explaining the etiology and specific phenomenological features of OCD. These theories aim to explain compulsion by patients having certain beliefs about danger, responsibility, and their thoughts in relation to their actions. Cognitive and metacognitive theories emphasize that cognitions either cause or enable compulsive behavior (Szalai, 2019). Although cognitive and metacognitive approaches do not clarify every aspect of OCD, they help to better understand the processes of development and maintenance of specific symptoms of the OCD. For instance, the overestimation of the significance of thoughts as well as an exaggerated feeling of responsibility for events in the world can account in part for an excessive sense of control. However, cognitive and metacognitive theories are unable to account for specific action-related features of OCD such as the feeling of incompleteness, the loss of motor control and agency, and the repetitiveness of compulsive behavior (Schmidt et al., 2021). To explain and understand these phenomenal and behavioral features of compulsive acts in OCD, several authors have argued for altered sensorimotor function or altered subjective experience of sensorimotor function (Gentsch et al., 2012; Levy, 2018; Szalai, 2019; Schmidt et al., 2021).

One promising sensorimotor approach provides theoretical explanation for these action related features in OCD and received some empirical support (Gentsch et al., 2012; Lazarov et al., 2012; Russo et al., 2014). This sensorimotor theory, the “seeking proxies for internal states” (SPIS) model, assumes that individuals with OC tendencies or clinically relevant symptoms have diminished conscious access to internal states such as specific emotions or proprioception and compensate for this deficiency by relying on observable proxies (Lazarov et al., 2010; Dar et al., 2021; Lazarov et al., 2024). According to the model, “proxies” are external confirmable signs of internal states (e.g., hunger, muscle tension, emotions, motivations, memories), used to make up for their lacking inner subjective experience (Dar et al., 2019). By relying on external proxies (e.g., environmental stimuli, procedures or rituals, rules, behaviors) the SPIS model assumes, that the function of repetitive behaviors in patients with OCD is to produce a sense of control and security, but, result in a vicious cycle of anxiety, loss of motor control, and a distorted sense of self and agency (Lazarov et al., 2010; Dar et al., 2021; Lazarov et al., 2024). For example, in checking compulsions, an individual experiencing obsessive-compulsive symptoms who fears setting the house on fire, may engage in specific rituals to ensure the stove is turned off, such as checking repeatedly, or taking a photograph of the stove knobs instead of relying on memory. Because the environment becomes the initiator and proxy of action initiation and completion rather than the individual, people with OCD may experience a diminished sense of control and agency (Oren et al., 2019). A sense of agency arises when the predicted outcomes of one’s actions correspond to the actual sensory consequences of those actions (Chambon et al., 2014). In OCD, this predictive matching process may be weakened due to a reduced reliance on internal feedback and increased dependence on external cues (“proxies”), leading individuals to experience their actions as less causally effective and less self-generated (Gentsch et al., 2012; Oren et al., 2019). However, there are some aspects that should be considered when investigating the putatively disturbed sense of agency in OCD patients. Attempts to explicitly capture and measure the phenomenological features associated with agency can be subject to biases (Haggard, 2017). Especially when compulsions are associated with beliefs of inflated responsibility, explicit agency judgements might reveal not more than the confirmation of these preconceived beliefs (Belayachi and Van der Linden, 2010; Gentsch et al., 2012).

Therefore, an experiment is needed that can provide additional insight into the subjective experience of action and agency through implicit measures. Such an investigation of the subjective experience of agency could be carried out by means of the temporal or intentional binding paradigm (Moore and Obhi, 2012). This paradigm measures the phenomenon called temporal binding effect. The temporal binding effect occurs under voluntary movement and links or binds awareness of a voluntary action with awareness of its sensory outcome, bringing them closer together in the subjectively perceived time (Haggard et al., 2002). A sense of agency is as well produced when there is a match between the predicted consequence of a movement and its actual sensory outcome, linking the two concepts of agency and temporal binding together (Moore and Obhi, 2012). It must be noted that, although the temporal binding paradigm has become a widely used implicit measure for the sense of agency, the exact nature of this relationship remains debated. A substantial body of research suggests that the presence of intentional action is a prerequisite for the occurrence of binding effects, as temporal shifts are typically observed only when outcomes follow voluntary actions and not when movements are externally generated or passively observed (Haggard et al., 2002; Humphreys and Buehner, 2010; Haggard, 2017). However, despite its robustness, the paradigm itself is not without criticism. More recent work proposes that temporal binding may only indirectly reflect the subjective experience of agency and arises from more general mechanisms of predictive processing or multisensory temporal integration without the necessity of intentionality (Weller et al., 2020; Gutzeit et al., 2023). From this perspective, agency related binding may represent a specific subtype within a broader class of temporal binding phenomena, rather than its causal origin (Gutzeit et al., 2023). In the present study, we follow the common practice of interpreting temporal binding as an implicit indicator of agency, while acknowledging that the underlying processes may not be exclusively agency driven. Therefore, a typical sense of agency is characterized by a binding pattern consisting of action binding and tone binding. If a tone is voluntarily elicited by one’s own action, for example by pressing a button, then one’s own action is perceived as being closer to the tone and the tone is perceived as being closer to the performed action, compared to when each is presented separately. Specifically, when tone and action are presented and judged in two separate conditions, no such shift in time perceptions occurs. The shift of the action toward the presented tone is called action binding. Is the tone in one’s time perception shifted closer to the action which initiated it, then this is called tone binding. Importantly, Caspar et al. (2016) showed that the binding effect is attenuated when the experimenter coerced participants into movement, i.e., depriving them of a free choice. This feeling of being compelled or even coerced to perform a movement is a decisive characteristic of compulsive behavior.

However, in the only study to date examining temporal binding and OC tendencies, Oren et al. (2019) provided evidence supporting the assumptions of the SPIS model. Oren et al. (2019) used the temporal binding task to test predictions of the SPIS model on the sense of agency, comparing 54 individuals with high and 48 with low OC tendencies (based on OCI-R scores). The authors introduced both congruent and incongruent tone conditions to disentangle predictive and retrospective components of temporal binding. In the congruent condition, the tone that followed an action was consistent with the participant’s expectation based on prior trials, whereas in the incongruent condition, the tone violated these expectations. This manipulation allowed the authors to test whether the observed binding effect depended on predictive processes or on retrospective, postdictive evaluation of the outcome. In the classical binding condition, participants with higher OC tendencies showed significantly weaker tone binding than those with lower OC tendencies, suggesting an attenuated implicit sense of agency. However, this task design could have introduced another factor that distorts the performance of the experiment and thus the results of the classic binding phenomenon. Moreover, the study focused exclusively on tone binding and did not investigate action binding, which limits the interpretation of the findings with respect to the full temporal binding phenomenon. In addition, Oren et al. did not report the OCI-R total scores or the specific cut-off values used to define high and low OC groups, making it difficult to directly compare the group characteristics and the magnitude of the effects reported. The current study aims to replicate Oren et al.’s findings while extending their work by investigating action binding in OC tendencies to further test the SPIS model.

Based on the SPIS model, individuals with higher OC tendencies may not exhibit the typical temporal binding pattern linked to an unaffected sense of agency, where actions and outcomes are perceived as occurring closer in time. Specifically, those with higher OC tendencies, potentially lacking access to internal states such as the timing of their actions (e.g., key presses), may rely on external cues (e.g., tones) to compensate (Schmidt et al., 2021). This could result in tones and key presses being perceived closer to the actual tone time compared to individuals with lower OC tendencies. Tone and action binding are measured as the difference between perceived timing in baseline and operant conditions, yielding a judgment error for comparison. In the higher OC group, we expect larger judgment errors in action binding and smaller errors in tone binding. Oren et al. (2019) also found a discrepancy between implicit (binding) and explicit (self-reported) agency measures, with higher OC participants showing weaker binding but higher self-reported control. This aligns with studies showing weak correlations between implicit and explicit agency measures (Kumar and Srinivasan, 2013; Dewey and Knoblich, 2014). However, many studies rely on non-standardized oral reports. To address this, we use the Sense of Agency Scale (Tapal et al., 2017) to systematically examine the relationship between implicit and explicit agency measures. This questionnaire distinguishes between two complementary components of explicit agency. First, the positive sense of agency, reflecting the feeling of being in control of one’s body, mind, and environment and second, the sense of negative sense of agency reflecting the experience that these domains are not under one’s control.

Thus, the goal of this study is to examine how the sense of agency manifests in healthy individuals with obsessive-compulsive tendencies. We aim to extend the study of Oren et al. (2019) by investigating differences in action binding and using the Sense of Agency Scale as a standardized measure of explicit agency. Based on the SPIS model, we hypothesize that individuals with higher OC tendencies will show a weaker tone binding effect and a stronger action binding effect. Additionally, we expect a discrepancy between implicit and explicit measures of agency. Furthermore, in addition to OC tendencies, exploratory analyses were conducted to examine potential associations between binding effects and broader affective traits such as anxiety, given the well-documented overlap between OC and anxiety symptoms (Goodwin, 2015; Gillan et al., 2017).

## 2 Materials and methods

### 2.1 Participants

Based on the original study by Haggard et al. (2002), we estimated a within-subject effect size for the temporal binding effect of d = 0.6. Comparable procedure and effect sizes have been reported by other studies (Humphreys and Buehner, 2010; Gutzeit et al., 2023). An *a priori* power analysis using G*Power (Version 3.1.9.6) was performed for the within-subject binding effect, with an estimated effect size of d = 0.6 (two-tailed paired t, *α* = 0.05). The analysis indicated that *n* = 27 participants are required to achieve 85% power. Accounting for an estimated 10% dropout rate due to technical problems, the target sample size was set to *n* = 30. Because Oren et al. (2019), did not report OCI-R scores, cut-offs, or group differences in action binding, an exact *a priori* power analysis was not feasible. The sample size was therefore based on the power analysis for the overall binding effect. Hence, our sample provides preliminary estimates of the relationship between OC tendencies and binding effects. A non-clinical sample was employed to allow for controlled investigation of the fundamental mechanisms underlying OC tendencies free from confounding effects of pharmacological medication, comorbid psychiatric conditions, and symptom heterogeneity often present in clinical OCD samples. Although this design limits the direct generalization of our findings to clinical OCD populations, it affords a clearer delineation of the cognitive mechanisms of agency that may contribute to the disorder. Thus, thirty German speaking healthy participants were recruited for this study from the local community of the Friedrich Schiller University, Jena, Germany. The Mini International Neuropsychiatric Interview (M. I. N. I; Sheehan et al., 1998) was conducted to ensure that the study participants did not fulfill any criteria for a current mental disorder according to DSM-5 criteria. Another exclusion criterion was a history of ADHD or a tic disorder, which was systematically assessed in the structured clinical interview. One participant was excluded from the study due to clinically significant OCD symptoms. Thus, the final sample consisted of 29 participants (55% females) and had a mean age of 24.1 years (SD = 3.4; range: 19–32). All participants reported normal hearing and normal or corrected vision. All participants underwent a series of neuropsychological tests and questionnaires. The local ethics committee of the Friedrich-Schiller University, Jena, Germany approved the study. Informed written consent was obtained from all participants before their participation.

### 2.2 Neuropsychological test

**Figure 1:**
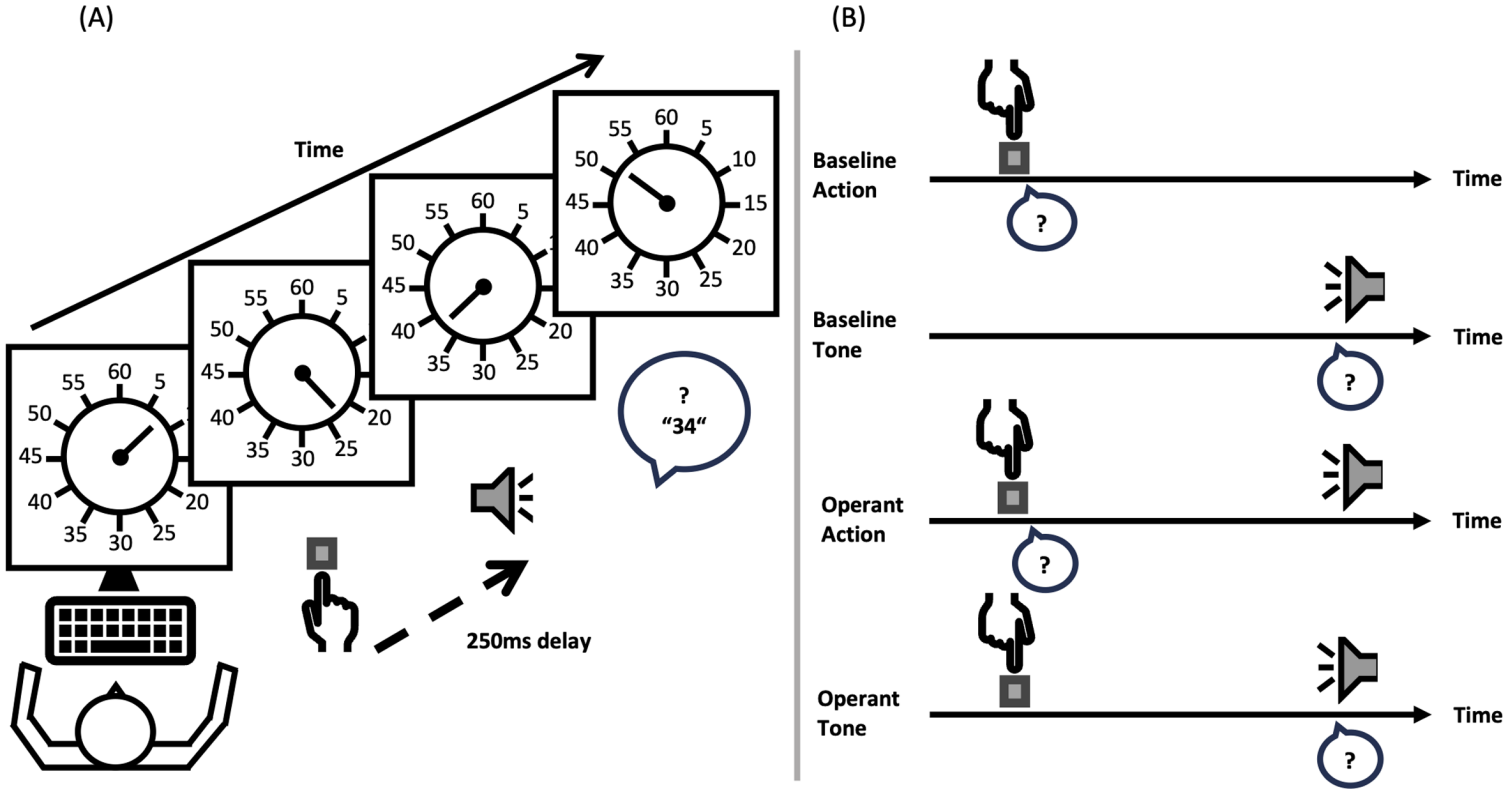
Setup temporal binding task. Depicted is the setup of the temporal binding task **(A)** and the four conditions **(B)** presented to participants in random order. The question marks in the speech bubbles indicate which event, keypress or tone, the participant should classify in terms of time perception. Watching the constantly rotating clock hand, the participant reports where the clock hand was when s/he pressed the button (baseline action or operant action) or heard the tone (baseline tone or operant tone).

The temporal binding paradigm is based on the classic psychophysical “Libet experiment” (Libet et al., 1983). The standard approach is extensively studied and applied by Haggard and colleges (Haggard et al., 2002; Haggard et al., 2003; Haggard, 2017). Participants watch a clock hand rotating at around 0.4 Hz (Figure 1).

The task consists of four different conditions. The four separated blocks are divided into two baseline conditions and two operant conditions. The order of the four blocks was randomized across participants to avoid practice or sequence effects. In one of the baseline conditions, the action only condition, participants have to press a key at a time of their own choice and report the clock hand position they perceived when they pressed the key. In the other baseline condition, the tone only condition, participants hear only a tone at some point during the clock hand rotation and report the clock hand position they perceived at the time they heard the tone. The two operant conditions require two time judgements. Participants press again a key at a time of their own choice but then hear also a tone 250 ms later. In one operant condition, they have to report the clock hand position at the time they perceived their key press. In the other operant condition, they report the clock hand position at the time they heard the tone. To obtain the binding effects, the time judgements from the operant condition are compared with the time judgements from the baseline condition. The difference between the conditions is sometimes referred to as judgement error (Oren et al., 2019). Specifically, action binding is calculated by measuring the difference between the time judgement in the action only baseline condition and the time judgement of the action in the operant condition. Tone binding, on the other hand, is calculated by measuring the difference between the time judgement in the tone only baseline condition and the time judgement of the tone in the operant condition. Every participant completed the temporal binding task under approximately the same standardized conditions.

### 2.3 Clinical assessment tools

A relationship between depression, anxiety and sense of agency has been shown (Hovenkamp-Hermelink et al., 2019). Therefore, in addition to the OCI-R, all participants were presented with the BDI-II, the STAI and the SoA scale.

The Obsessive-Compulsive Inventory-Revised (OCI-R; Foa et al., 2002) consists of 18 items describing obsessive-compulsive symptoms (e.g., “I repeatedly check doors, windows, drawers, etc.”) in the form of self-reports, which are rated on a 5-point Likert-scale from 0 (not at all) to 4 (extremely) according to how much impairment or suffering they caused in the past month. Total scores range from 0 to 72, with higher scores reflecting greater impairment or suffering caused by obsessive-compulsive symptoms. The OCI-R has been shown to have good test–retest reliability, validity, and overall excellent internal consistency (*α* = 0.88; Hajcak et al., 2004) in both clinical (Foa et al., 2002) and non-clinical samples (Hajcak et al., 2004).

The State–Trait Anxiety Inventory (STAI; Spielberger et al., 1983) is a self-report measurement of anxiety and consists of two 20-item subscales, evaluating the current state of anxiety (e.g., “I am nervous”) as well as the relatively stable aspects and general states of anxiety (e.g., “I worry too much over something that really does not matter”). Items are rated on 4-point Likert-scales from 1 (not at all) to 4 (very much so) and total scores range from 20 to 80 for each subscale, with higher scores reflecting greater state or trait anxiety. The STAI is a well-established and frequently administered instrument with excellent internal consistency (α = 0.90; Spielberger et al., 1983; Barnes et al., 2002).

The Beck Depression Inventory-II (BDI-II; Beck et al., 1996) is a self-report tool consisting of 21 items and measuring depressive severity (e.g., “I am sad all the time”), rated from 0 to 3 based on symptom intensity over the past 2 weeks. Total scores range from 0 to 63, with higher scores reflecting greater depressive severity. The scale has demonstrated excellent internal consistency (α = 0.90; Wang and Gorenstein, 2013).

Furthermore, in addition to the implicit measurement of sense of agency through the temporal binding task, an explicit assessment was also sensitive enough to show an altered sense of agency in individuals with OC tendencies (Tapal et al., 2017). One such explicit measurement of agency is the Sense of Agency Scale. Established by Tapal et al. (2017) and originally in Hebrew translated into English, the Sense of Agency Scale is a direct measurement to assess a person’s general beliefs about his/ her agency. It consists of an overall scale, often referred to as global sense of agency, and two subscales, the sense of negative agency (SoNA) (e.g., “I am just an instrument in the hands of somebody or something else”) and the sense of positive agency (SoPA) (e.g., “I am in full control of what I do”). Participant’s responses are recorded on a scale from 1 (strongly disagree) to 7 (strongly agree), with a total of 13 items. Higher global SoA scores, ranging from 13 to 91, indicate a stronger overall belief in one’s personal control and causal influence in the world. The two subscales capture complementary facets of explicit agency. The SoPA, ranging from 7 to 49, reflects the experience of being in control of one’s body, mind, and environment, with higher scores indicating a stronger perceived sense of control, whereas the SoNA, ranging from 6 to 42, reflects the perception that these domains are not under one’s control, with higher scores indicating stronger feelings of lack of control. The questionnaire was translated from English into German by cross-validation. The cross-validation included translating the scale into German, back-translating it and checking its accuracy through an expert exchange (G. W., S. S.). A validated German version of the Sense of Agency Scale has since been published (Bart et al., 2023). However, as data collection for the present study was completed prior to its publication, this version was not yet available. Upon comparison, our translation closely corresponds to the validated German version in item wording and meaning, and the only conceptual difference identified was that the German version of Bart et al. (2023) consisted of only 11 items. Item 4 (“I am the author of my action”) as part of the SoPA subscale and Item 5 (“The consequences of my actions feel like they do not logically follow my actions”) as part of the SoNA subscale are included in the version we adapted from Tapal et al. (2017) but are exclude in the German version of Bart et al. (2023). This should be considered when scoring the scales and comparing the total scores between studies. The 2-month test–retest reliability of the original 13-item scale translated and used here was *r* = 0.78 for the SoPA and *r* = 0.74 for the SoNA and the internal consistency was *ω* = 0.78 for the SoPA and ω = 0.76 for the SoNA (Tapal et al., 2017). Global sense of agency as well as SoPA and SoNA scores were calculated.

All participants in the present study completed the OCI-R, STAI, BDI-II, and SoA scale.

### 2.4 Statistical analyses

The statistical analyses of binding effects are based on the judgement errors. Judgment errors were calculated by subtracting the time difference between the actual occurrence of an event and participants’ judgments, based on their reports of the clock’s position (Tapal et al., 2017). This was followed by subtracting each subject’s mean judgment error in the single-event baseline conditions (i.e., when keypress and tone were presented separately) from the mean judgment error for the same event in operant conditions (i.e., when keypress and tone were presented together). This allowed for the measurement of perceptual shifts that indicate the binding between voluntary actions and their subsequent effects. To test whether these binding effects were present, we used one-tailed t-tests against zero to test for the presence of binding effects, expecting positive shifts for action binding (“greater than zero”) and negative shifts for tone binding (“less than zero”).

In order to explain the variance in the binding effects, a stepwise linear regression analysis was carried out. Subsequently, we performed a median split based on the OCI-R total score to investigate the relationship between action binding, tone binding and OC tendencies, establishing a lower OC tendency group (LOC) and higher OC tendency group (HOC). Tone binding as well as action binding were compared between both groups using a Student’s t-test. Furthermore, Sense of Agency Scale scores were compared between both groups using a Welch’s t-test. To explore the relationship between the dependent variable, i.e., the binding effects, and a set of potential predictor variables in the total group as well as in the HOC and LOC groups we employed a stepwise linear regression using a forward selection approach. Significance level for all statistical analyses was set at *α* = 0.05.

## 3 Results

### 3.1 Total sample

The total sample consisting of 29 participants showed an OCI-R mean score of *M* = 6.14 (SD = 4.61).

#### 3.1.1 Implicit sense of agency – significant binding effects

The analysis of the binding effects in the whole sample revealed significant findings for action (*M* = 24.81, SD = 38.14, t (28) = 3.50, *p* = 0.002, *d* = 0.65) and tone binding (*M* = −52.70, SD = 72.52, t (28) = −3.91, *p* < 0.001, *d* = −0.73). Both were significantly different from zero, indicating the presents of a tone and action binding effect.

#### 3.1.2 Explaining binding effects – regression analysis

**Figure 2:**
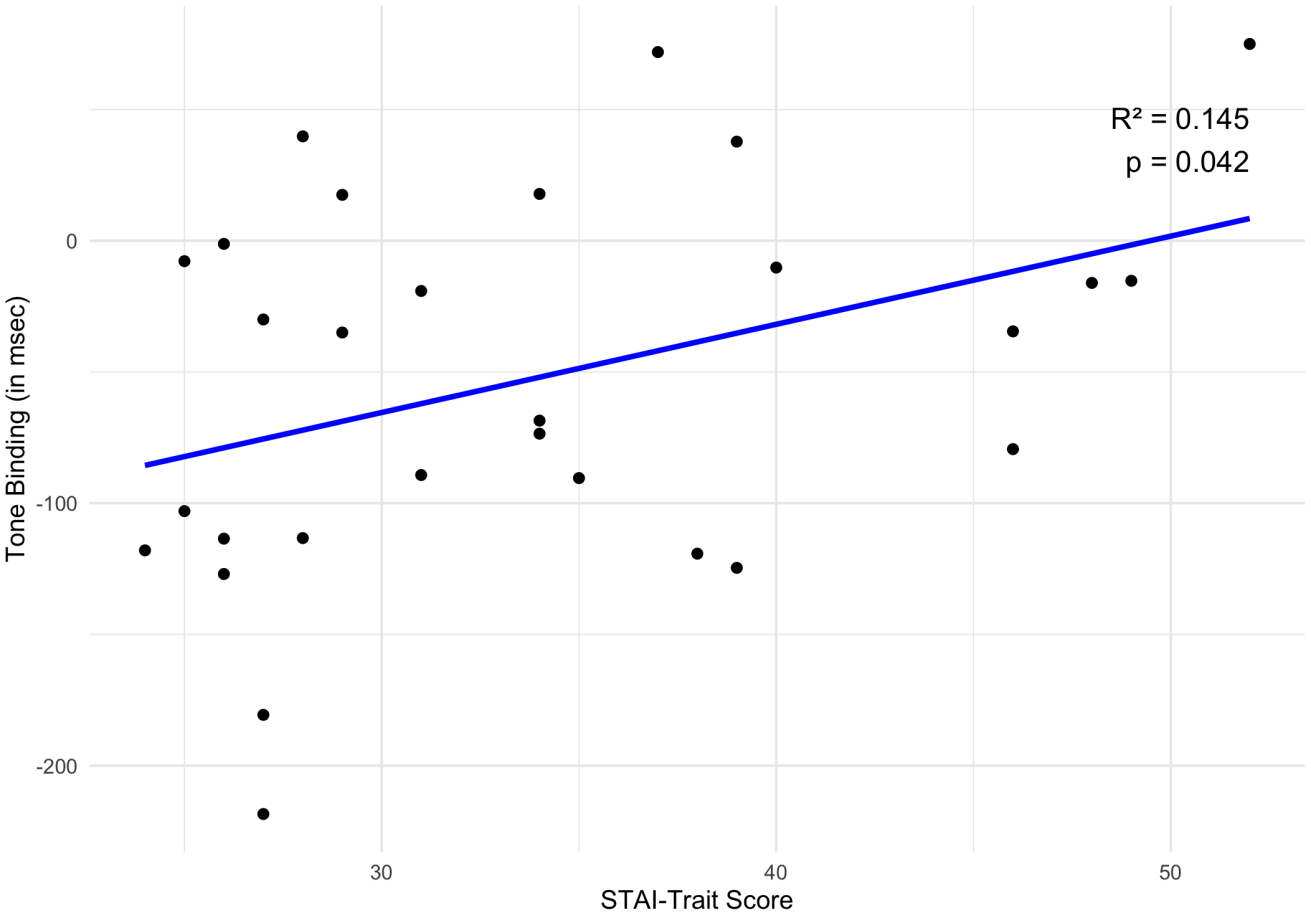
Regression analysis: the relationship between tone binding and STAI trait in the total sample. Scatter plot illustrating the relationship between STAI-Trait scores (*x*-axis) and tone binding (in milliseconds; *y*-axis). Each point represents an individual participant’s data, with more negative tone binding values indicating a stronger binding effect. A linear regression line is superimposed, revealing a statistically significant positive association (*R*^2^ = 0.145, *p* = 0.042). Thus, higher STAI-Trait scores are linked to less negative (i.e., weaker) tone binding values.

In an exploratory stepwise regression analysis, only the STAI trait component (beta = 3.36, t = 2.14, *p* = 0.042) explained significantly the tone binding effect (*F* (1, 27) = 4.56, *R*^2^ = 0.145, *p* = 0.042), as depicted in Figure 2. Action binding in the total group was not significantly explained by any of the included variables.

### 3.2 Testing differences between lower OC tendency group vs. higher OC tendency group

The HOC group consisted of *n* = 15 participants with a mean OCI-R score of *M* = 9.73 (SD = 3.31) and the LOC group consisted of *n* = 14 participants with a mean OCI-R score of *M* = 2.29 (SD = 1.77).

#### 3.2.1 Differences in the implicit sense of agency

**Figure 3:**
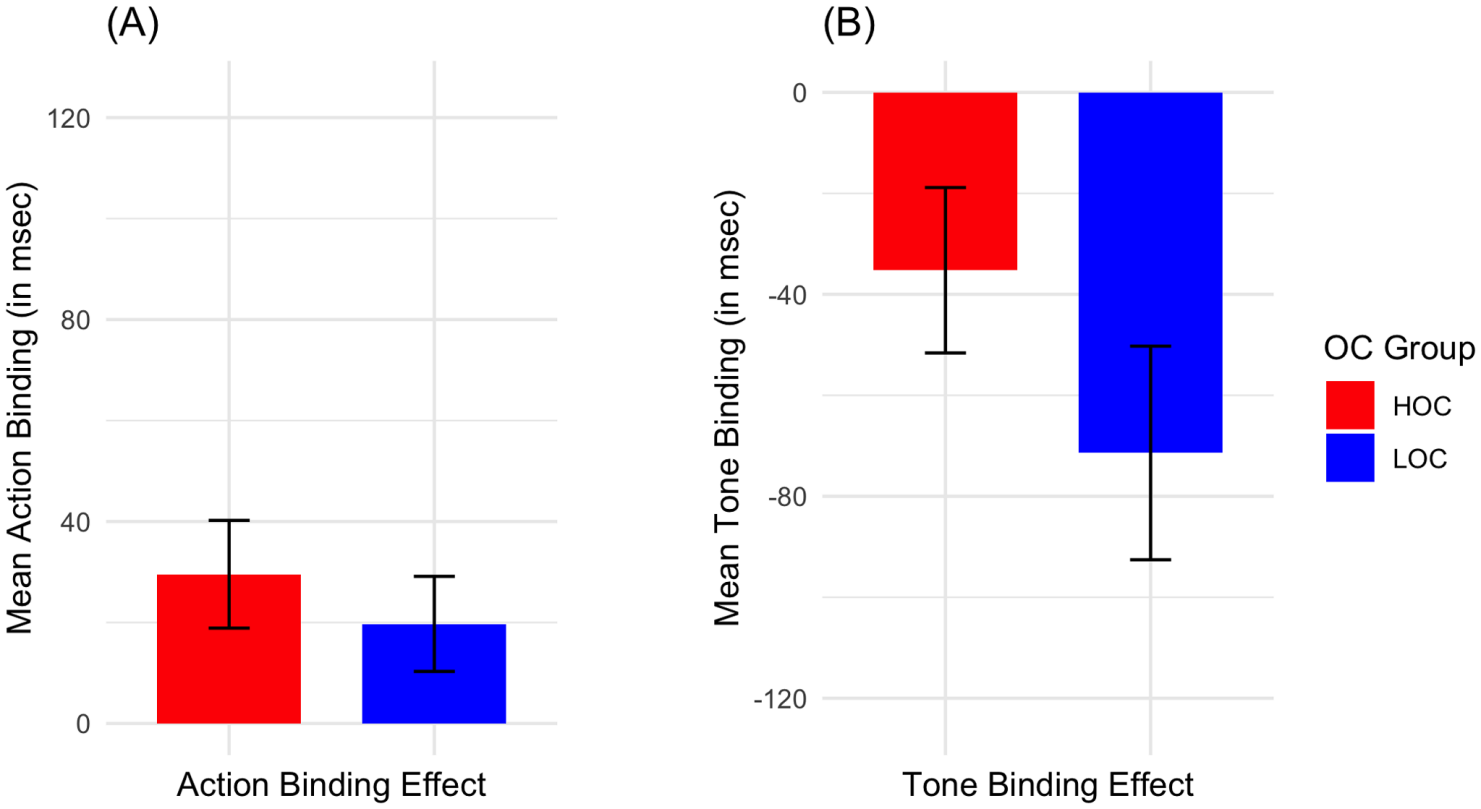
Comparison of tone binding and action binding effects between LOC and HOC groups. Illustrated are the mean action binding **(A)** and tone binding **(B)** effects for the LOC (blue) and HOC (red) groups. The bars represent group averages, while the error bars indicate the standard error of the mean (SEM). The action binding effect **(A)** is shown as positive mean values on the *y*-axis, with higher values reflecting stronger binding. Conversely, the tone binding effect **(B)** is shown as negative mean values, with lower values indicating stronger binding. Both groups show significant binding effects, although, group comparison revealed no significant group differences in action and tone binding.

The mean action binding in both the HOC group (*M* = 29.56, SD = 41.33, t (14) = 2.77, *p* = 0.008, *d* = 0.72) and the LOC group (*M* = 19.73, SD = 35.21, t (13) = 2.1, *p* = 0.028, *d* = 0.56) was significantly greater than zero. The mean tone binding in both the HOC group (*M* = −35.24, SD = 63.43, t (14) = −2.15, *p* = 0.025, *d* = −0.56) and the LOC group (*M* = −71.42, SD = 79.14, t (13) = −3.38, *p* = 0.002, *d* = −0.90) was also significantly greater than zero. The mean values are depicted in the Figure 3.

When comparing the mean binding effects between both groups with a Student’s t-test, no significant differences were found for the tone binding (t (27) = −1.36, *p* = 0.184, *d* = −0.51) and action binding (t (27) = −0.69, *p* = 0.498, *d* = −0.26) effects indicating a similar sense of agency for HOC and LOC. A post-hoc sensitivity analysis indicated that the achieved power to detect between-group differences of the magnitude observed was low. With the present subgroup sizes, power to detect the observed between-group difference in tone binding (*d* = 0.51) was approximately 26%, and power to detect the observed difference in action binding (*d* = 0.26) was approximately 10%.

#### 3.2.2 Differences in explicit sense of agency

Using the Welch’s t-test, we found that the HOC group (*M* = 78.07, SD = 8.26) reported a significantly lower overall explicit sense of agency than the LOC group [(*M* = 84.00, SD = 4.08), t (20.74) = −2.48, *p* = 0.022, *d* = −0.91]. Further comparisons of the Sense of Agency Scale subscales revealed that the HOC group (*M* = 34.53, SD = 4.39) had a significantly lower sense of positive agency than the LOC group [(*M* = 37.64, SD = 2.74), t (22.33) = −2.37, *p* = 0.027, *d* = −0.87]. In contrast, the HOC group (*M* = 12.48, SD = 5.06) showed a trend toward a higher sense of negative agency compared to the LOC group [(*M* = 9.64, SD = 2.87), t (22.47) = 1.87, *p* = 0.075, *d* = 0.69].

#### 3.2.3 Explaining implicit sense of agency – regression analysis

**Figure 4:**
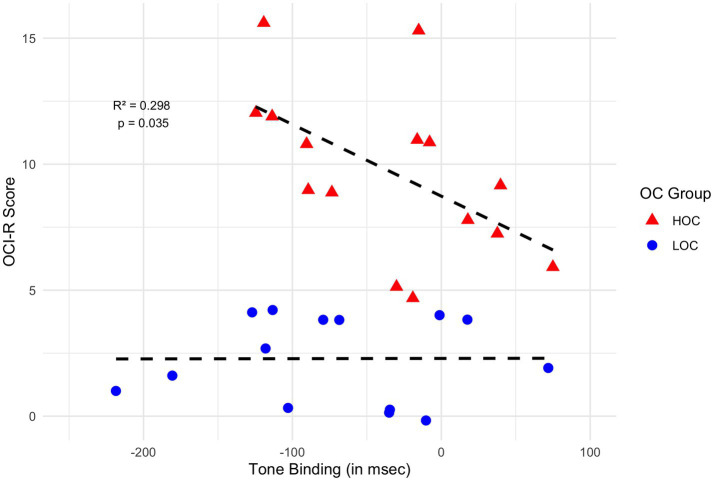
Correlation between tone binding and OCI-R scores for the HOC and LOC groups. Scatter plot displays the relationship between tone binding (in milliseconds, *y*-axis) and OCI-R (*x*-axis) scores for participants in the HOC (red) and LOC (blue) group. Data points are jittered along the x-axis to reduce overplotting. The dashed regression line represents the linear fit for each group. The fitted regression line reveals a significant negative association (*R*^2^ = 0.298, *p* = 0.035) between OCI-R scores and tone binding in the HOC group, indicating that higher OCI-R scores predict more negative tone binding values and, thus, a stronger tone binding effect with increasing OCI-R scores. The regression analysis in the LOC group revealed no significant association between OCI-R scores and tone binding.

Tone binding in the HOC group was significantly predicted by OCI-R total scores (*F* (1, 13) = 5.53, *R*^2^ = 0.298, *p* = 0.035; beta = −10.49, *t* = −2.35, *p* = 0.035). The relationship between OCI-R scores and tone binding is illustrated in Figure 4. In contrast, action binding in the HOC group was not significantly influenced by any variables. Additionally, no significant effects of obsessive-compulsive, depressive, or anxiety tendencies on action or tone binding were observed in the LOC group.

## 4 Discussion

The goal of the present study was to investigate the relationship between the sense of agency in a group of healthy subjects with OC tendencies. Based on SPIS model, we hypothesized an attenuated implicit sense of agency in the group with high OC tendencies, expressed by a diminished tone binding effect, but heightened action binding effect.

In line with expectations, our findings revealed significant action and tone binding effects in the entire sample, indicating a perceived temporal closeness between voluntary actions and their sensory outcomes. These results fit well with the results of other studies (Haggard et al., 2002; Oren et al., 2019) and support the general idea of the existences of a temporal binding effect, highlighting the need for further investigation. Significant binding effects persisted following the classification into higher and lower OC tendency groups, suggesting that temporal binding is preserved even in individuals with higher OC tendencies.

However, contrary to our hypotheses and the assumptions of the SPIS model, we did not find statistically significant differences in action and tone binding effects between the HOC and LOC groups. These findings suggest a normal implicit sense of agency in individuals with higher OC tendencies. Nonetheless, it is important to note that the group comparison was based on relatively small subgroups, which limits statistical power. The *a priori* power analysis based on the original within-subject binding effects reported by Haggard et al. (2002) indicated that the total sample size was sufficient to reproduce the robust within-subject binding effects. In contrast, our post-hoc sensitivity analysis showed that with the current sample, the between-group analyses were underpowered. Accordingly, the nonsignificant group comparisons should be interpreted as inconclusive and regarded as preliminary estimates, rather than as strong evidence for the absence of such effects. Yet, our findings add new evidence to the literature by examining and showing a preliminary intact binding effects in HOC individuals compared to LOC individuals. Nonetheless, our results are also in contrast with the study by Oren et al. (2019), who did not account for action binding but only examined tone binding. Oren et al. (2019) found a significant lower tone binding effect in participants with higher compared to lower OC tendencies, indicating an attenuated implicit SoA in this group. Three reasons could be responsible for the different results between this and Oren’s study. First, although temporal binding is a well-established method of capturing the sense of agency, it is yet subject to methodological differences, i.e., task explanation, experimental setup, and analysis of binding effects. Oren et al. (2019), for example, may have introduced additional cognitive demands by presenting incongruent and congruent tones, thereby influencing participants’ temporal judgments and overall task performance. Furthermore, Oren et al. (2019) excluded trials in which the judgment error was larger than 5 s arguing that in those trials participants might have not paid sufficient attention. Second, Oren et al. (2019) as well as our results show a large standard deviation of the mean binding values suggesting high individual differences in task performance and, thus, making generalizations and conclusions drawn from the task less reliable. This is especially important when considering the differences in simple size between our and Oren et al. (2019) study. As discussed above, although the observed effect sizes in our study were comparable to those reported by Oren et al. (2019), our sample size was likely too small to detect such effects at a statistically significant level. Considering the large variability typically observed in temporal binding paradigms, the task may lack sufficient sensitivity to reliably capture subtle group differences in smaller samples. Therefore, replication in a larger cohort is essential to determine whether the observed trends reflect true effects or sample related variability. Third, OC tendencies might not only differ in symptom clusters due to the heterogenic nature of OC behavior, but also in the degree of symptom severity. Where we chose the median split method to divide our sample into HOC and LOC, Oren et al. (2019) chose the lower and upper 25% of the distribution of the OCI-R scores for sampling. Furthermore, Oren et al. (2019) also did not report the OCI-R total scores making it difficult to compare the investigated groups. Especially in a condition as heterogeneous as OCD and here OC tendencies it is crucial to account for sample characteristics and symptom dimensions. Given that obsessive-compulsive features are also transdiagnostically related to other affective and anxiety traits, controlling for comorbidity and dimensional variation is essential when interpreting group differences. This is particularly relevant for implicit measures such as temporal binding, which likely emerge from early perceptual and sensorimotor processes that may differ across OC symptom clusters. For instance, checking-related OC tendencies may involve distinct cognitive mechanisms and uncertainty monitoring processing compared to washing-related tendencies, potentially influencing temporal binding in different ways. This must be account and controlled for in future studies.

In addition, an exploratory regression analyses revealed that, across the entire group, tone binding was significantly predicted by trait anxiety levels but not by OC symptom severity. The absence of an association with OC symptom severity may reflect the restricted range of OCI-R scores in our non-clinical sample and that such relationships emerge only at higher, clinically relevant levels of symptom expression. In contrast, anxiety may exert a broader transdiagnostic influence on temporal binding through heightened uncertainty and altered predictive processing, partially overlapping but not identical to mechanisms implicated in OC tendencies. This *post hoc* finding suggests that higher trait anxiety is associated with a reduced tone binding effect, potentially indicating an attenuated sense of agency. Trait anxiety, distinct from state anxiety, reflects a persistent tendency to experience fear and worry across various situations over time (Knowles and Olatunji, 2020). Although this relationship was not part of our *a priori* hypotheses, it is consistent with previous literature linking elevated trait anxiety to altered perceptions of control (Song and Li, 2019), particularly regarding the causal attribution of events and, thus, linking it to the sense of agency. Specifically, trait anxiety has been found to be positively correlated with the concept of external locus of control (Song and Li, 2019). The concept of locus of control refers to the “generalized attitude, belief, or expectancy regarding the nature of the causal relationship between one’s own behavior and its consequences” (Rotter, 1966). It is a unidimensional concept assessing individuals’ perceived ability to control events between high external and high internal locus of control. Individuals with a higher external locus of control attribute events to external factors (e.g., chance, fate), while those with a higher internal locus of control perceive events as dependent on internal factors (e.g., abilities, effort). By definition, an internal locus of control aligns with a stronger sense of agency, whereas an external locus of control corresponds to a weaker sense of agency. This link may explain our findings. Consistent with the SPIS model’s assumptions in OCD and OC tendencies, higher trait anxiety appears to shift focus from internal factors (e.g., actions) to external proxies (e.g., tones). Specifically, individuals with elevated trait anxiety may perceive less control over events, such as tone occurrences, attributing them instead to chance or coincidence rather than as a result of their own actions. This shift alters their temporal perception of the tone, resulting in an attenuated sense of agency. Nevertheless, this result warrants cautious interpretation and should be considered preliminary pending replication in studies specifically designed to investigate the influence of trait anxiety on temporal binding.

However, a study by Tobias-Webb et al. (2017) found no association between implicit measures of sense of agency and locus of control. Further research is needed to better understand the relationship between these constructs.

Although neurocognitive mechanisms such as action–outcome temporal delay and dopaminergic modulation have been implicated in temporal binding (Rammsayer, 1993; Rammsayer, 1999; Ruess et al., 2017) these factors were not directly assessed in the present study. Nevertheless, they provide a useful framework for interpreting our findings and for designing future studies. The short action–tone interval (250 ms) used here, likely engaged primarily subcortically driven timing mechanisms rather than cortically mediated executive processes (Rammsayer, 1993; Rammsayer, 1999), which may partly explain the robust within-subject binding effects observed across participants and across temporal binding studies. Prior research has also shown that dopaminergic activity modulates interval perception and binding strength (Rammsayer, 1993). In schizophrenia, linked to increased subcortical dopaminergic activity, temporal hyperbinding was observed (Haggard et al., 2003), whereas in major depressive disorder, possibly characterized by reduced mesolimbic/mesocortical dopamine, reduced temporal binding has been reported (Vogel et al., 2024). Taken together, subcortically mediated short interval binding may become more apparent in clinical populations with relatively known neurotransmitter changes. Besides our modest sample size for group comparison, this may also help to partly explain why, in our non-clinical sample with relatively mild OC tendencies, no clear group differences in temporal binding emerged and why replication in clinical OCD samples is warranted.

No study to date has examined temporal binding in OCD. Although action binding in our sample was not explained by any independent variables—possibly due to low variability in OCI-R scores—tone binding in the HOC group revealed that OC tendencies significantly predict temporal binding. Specifically, the OCI-R total score explained variance in tone binding, with higher obsessive-compulsive symptom levels associated with stronger (i.e., less negative) tone binding effects. This finding challenges the SPIS model’s assumption of diminished tone binding in individuals with higher OC tendencies. The SPIS model suggests that tone binding is attenuated because the tone serves as a proxy for time judgment. However, in our sample, tone binding increased with higher OC tendencies, contrary to the model’s predictions. This discrepancy warrants further investigation, particularly in clinical OCD populations.

Surprisingly, action binding in the HOC group was neither explained by any of the used clinical variables. Contrary to the assumptions of the SPIS model, this effect seems to be independent of OC tendencies, depressive tendencies or anxiety in the HOC group. However, this could change with a more pronounced manifestation of OC symptoms. Indeed, evidence exists for altered temporal binding in conditions such as major depressive disorder (Vogel et al., 2024), but not clearly in anxiety disorders or OCD. Further studies are required to test the results and trends found here. Our study has clearly shown that there is a need to investigate the temporal binding effect in OCD and anxiety disorder and that differences to healthy controls, especially in tone binding, are to be expected.

Finally, although, the binding patterns in our sample provide evidence for an intact sense of agency in individuals with higher OC tendencies when measured implicitly, we found, as predicted, a discrepancy between explicit and implicit measurements. Individuals with higher OC tendencies show a significant lower global sense of agency than individuals with lower OC tendencies, composed of a significant lower sense of positive agency and a higher sense of negative agency at trend level. The positive sense of agency reflects the experience of being in control of one’s thoughts, actions, and environment, whereas the negative sense of agency captures the perception that these domains are not under one’s control (Tapal et al., 2017). From this perspective, the present findings suggest that individuals with higher OC tendencies experience a weaker general feeling of control and are more prone to perceive their actions or outcomes as externally determined. Importantly, the use of the Sense of Agency Scale extends previous work by providing a standardized and psychometrically validated assessment of explicit agency beliefs. Although the current study focused on a non-clinical sample, these results may have implications for understanding OCD more broadly. Patients with OCD frequently report diminished feelings of control and heightened doubt regarding their own actions, consistent with an attenuated sense of agency. Thus, similar mechanisms underlying altered explicit agency beliefs in subclinical OC tendencies may also contribute to the cognitive and phenomenological disturbances characteristic of clinical OCD. However, to substantiate this assumption, future studies specifically examining agency processes in clinical OCD populations are needed. Furthermore, and in line with other studies (Kumar and Srinivasan, 2013; Dewey and Knoblich, 2014), explicit and implicit measurements of sense of agency were uncorrelated, indicating that the two concepts possibly measuring different aspects of the sense of agency. This difference between implicit and explicit sense of agency was also detected by Oren et al. (2019). However, their findings are based solely on directly questioning participants about their perceived control in the task, making it difficult to compare both findings. The difference between explicit and implicit sense of agency is widely discussed in the literature (Tapal et al., 2017). Oren et al. (2019) provide a possible explanation by arguing that implicit and explicit measurements might be subject to two different underling processes. However, the discrepancy can also be interpreted within the framework of the SPIS model (Lazarov et al., 2010; Dar et al., 2021), which posits that individuals with OC tendencies rely more heavily on external cues or ‘proxies’ to compensate for diminished access to internal states. While the implicit temporal binding task captures low level sensorimotor integration processes that seem to remain relatively preserved in our OC tendencies sample, the explicit Sense of Agency Scale requires reflective, belief-based judgments about one’s own control over thoughts and actions. Such explicit evaluations are more susceptible to cognitive biases related to perceived responsibility and the explicit attempt to create a sense of certainty possibly already observable in OC tendencies (Szalai, 2019). Therefore, the observed dissociation between implicit and explicit agency in our study may reflect a divergence between preserved sensorimotor agency processing and altered higher-order, conceptual representations of control.

Furthermore, it is possible that the temporal binding task only captures the sense of agency to a limited extent, as it reflects a temporal binding effect rather than intentionality per se (Wen and Imamizu, 2022). Empirical evidence suggests that the occurrence of binding depends critically on the presence of an intentional action and temporal shifts are observed when participants voluntarily initiate an action but not when the same movement occurs passively or is externally generated (Haggard et al., 2002; Wohlschlager et al., 2003; Humphreys and Buehner, 2010). This supports the view that binding is closely related to processes underlying agency attribution and action-effect learning. However, recent research suggests that intentionality may not be the sole or even primary mechanism underlying the temporal binding effect, which may instead emerge from broader processes of temporal perception and causal expectations (Weller et al., 2020; Gutzeit et al., 2023). In line with these perspectives, binding effects might depend primarily on the temporal and causal proximity between actions and outcomes, rather than on the subjective experience of agency itself (Suzuki et al., 2019). Consequently, while the temporal binding paradigm remains a valuable tool for studying the implicit aspects of agency, its interpretation should be made with caution, recognizing that it may reflect a broader class of temporal binding phenomena rather than a pure measure of intentionality.

### 4.1 Implications and limitations

Our study enhances the understanding of phenomenological aspects of compulsive behavior. However, several limitations should be noted. Although the total sample size was sufficient to detect within-subject binding effects, the smaller subgroup sizes resulting from the median split reduced statistical power to identify between-group differences. Our sample size was relatively modest and may have been underpowered to detect subtle effects or interactions between OC tendencies and both binding measures. The findings therefore provide preliminary estimates of this relationship that should be confirmed in larger, well-powered studies. Additionally, we focused on healthy individuals with subclinical OC symptoms, and while subgroup differences in OCI-R scores were significant, individuals with OCD may show different patterns in implicit and explicit measures of agency. A distorted sense of agency might vary with symptom severity and type (e.g., checking, washing). Thus, it is crucial to test our hypotheses in clinical OCD populations and further explore the relationship between temporal binding and specific OCD symptom clusters.

### 4.2 Conclusion

In conclusion, our study provides empirical support for a intact implicit sense of agency in individuals with obsessive-compulsive tendencies. The temporal binding paradigm served as a robust tool to investigate the intricate relationship between action binding, tone binding, and obsessive-compulsive symptoms. Further research, incorporating larger samples and subjects with clinically relevant symptoms, will help refine our understanding of the complex interplay between sensorimotor function and OCD.

## Data Availability

The raw data supporting the conclusions of this article will be made available by the authors, without undue reservation.
